# Isaridin E Attenuates Ischemic Brain Injury in Mice by Concurrently Reducing Platelet Hyperactivity and Microglial Neuroinflammation Associated with LRP1

**DOI:** 10.3390/md24090328

**Published:** 2026-09-20

**Authors:** Yu-Chen Mi, Zi-Cheng Li, Qi-Qi Jiang, Lan Liu, Sen-Hua Chen, Ni Pan, Guan-Lei Wang

**Affiliations:** 1 Department of Pharmacology, Zhongshan School of Medicine, Sun Yat-sen University, Guangzhou 510080, China; miych@mail2.sysu.edu.cn (Y.-C.M.); lzc3@outlook.com (Z.-C.L.); jiangqq7@mail2.sysu.edu.cn (Q.-Q.J.); 2 School of Marine Sciences, Sun Yat-sen University, Zhuhai 519082, China; cesllan@mail.sysu.edu.cn (L.L.); chensenh@mail.sysu.edu.cn (S.-H.C.); 3 Southern Marine Sciences and Engineering Guangdong Laboratory (Zhuhai), Zhuhai 519000, China; 4 Department of Pharmacy, The Second Clinical College, Guangzhou Medical University, Guangzhou 510261, China

**Keywords:** Isaridin E, ischemic stroke, platelets, microglial activation, neuroinflammation

## Abstract

Isaridin E (ISE), a marine-derived fungal cyclodepsipeptide, possesses antiplatelet activity in vitro and antithrombotic efficacy in a FeCl_3_-induced carotid artery thrombosis mouse model without prolonging bleeding time. In this study, we investigated the antiplatelet and neuroprotective effects of prophylactic and therapeutic administration of ISE in a transient middle cerebral artery occlusion (tMCAO) mouse model. We found that both prophylactic administration (50 and 100 mg/kg) and therapeutic administration (50 mg/kg) of ISE significantly reduced cerebral infarct volume, alleviated neurological deficits, and attenuated neuronal injury. Furthermore, therapeutic administration of ISE suppressed platelet hyperactivity, as evidenced by reduced P-selectin expression, downregulated platelet secretion-related proteins (SNAP23, VAMP8), and decreased plasma levels of platelet-derived pro-inflammatory cytokines (IL-1β, PF4, and CCL5). Moreover, ISE attenuated microglial inflammatory activation in the peri-infarct region in vivo. Mechanistically, in vitro studies revealed that this effect may be mediated through the LRP1/IκB/NF-κB signaling pathway. In conclusion, these results suggest that ISE protects mice against ischemic stroke by concurrently suppressing platelet hyperactivity and LRP1-associated microglial neuroinflammation.

## 1. Introduction

Ischemic stroke remains a leading cause of mortality and disability worldwide, accounting for approximately 87% of all stroke cases [1]. Despite advances in the understanding of ischemic stroke pathophysiology, intravenous thrombolysis with tissue plasminogen activator (tPA) remains the only approved pharmacological intervention for acute ischemic stroke [2]. However, this approach is restricted to a narrow therapeutic time window, strict eligibility criteria, and the risk of hemorrhagic transformation [2]. Therefore, novel therapeutic agents with an extended time window, broader applicability, and reduced bleeding risk are urgently needed for ischemic stroke treatment [3].

Beyond their well-established role in hemostasis, platelets are increasingly recognized as pivotal regulators of post-ischemic neuroinflammation [4,5]. Upon activation, platelets release a repertoire of pro-inflammatory mediators—including PF4, IL-1β, and CCL5—that can traverse the compromised blood–brain barrier and act as paracrine signals to prime microglia, the resident immune cells of the central nervous system [6,7,8]. Microglial activation represents a central pathological event following cerebral ischemia/reperfusion (I/R) injury [9]. While transient microglial activation may facilitate debris clearance and tissue remodeling, sustained or excessive inflammatory activation exacerbates blood–brain barrier disruption, neuronal apoptosis, and secondary brain injury [9,10]. Consequently, interrupting the propagation from platelet hyperactivity to microglia-mediated neuroinflammation represents a promising therapeutic strategy. Experimental studies have shown that targeting platelet GPVI (a key receptor for platelet adhesion and activation), or the vWF-GPIbα axis (the main mediator of initial platelet adhesion), but not GPIIb/IIIa, protected against ischemic brain injury by attenuating platelet-mediated inflammatory responses [11,12,13,14]. Despite the increasing diversity of antiplatelet drugs, most of them mainly target platelet aggregation and are limited by the bleeding risk, for instance, aspirin and clopidogrel [15,16]. Therefore, novel antiplatelet drugs that concomitantly suppress platelet hyperactivity and microglial inflammatory activation without increasing bleeding risk are urgently needed to improve clinical outcomes in acute ischemic stroke [17].

Marine-derived natural compounds, by their unique structure, have emerged as promising candidates for the development of antiplatelet and antithrombotic drugs [18]. Our recent study demonstrated that ISE, a cyclodepsipeptide isolated from the marine-derived fungus *Amphichorda felina* (syn. *Beauveria felina*) SYSU-MS7908 sourced from the South China Sea, possesses antiplatelet activity against ADP-induced platelet activation, secretion and aggregation in vitro and antithrombotic effects in an FeCl_3_-induced mouse carotid model in vivo, without prolonging bleeding time [19]. However, whether ISE exerts protective effects on ischemic stroke remains unclear. In this study, we used an adult mouse tMCAO model to evaluate the effects of ISE on post-stroke injury and to explore its potential mechanisms involving the suppression of platelet hyperactivity and microglial-mediated neuroinflammation.

## 2. Results

### 2.1. ISE Protected Against Brain Injury After Transient Cerebral Ischemia

**Figure 1:**
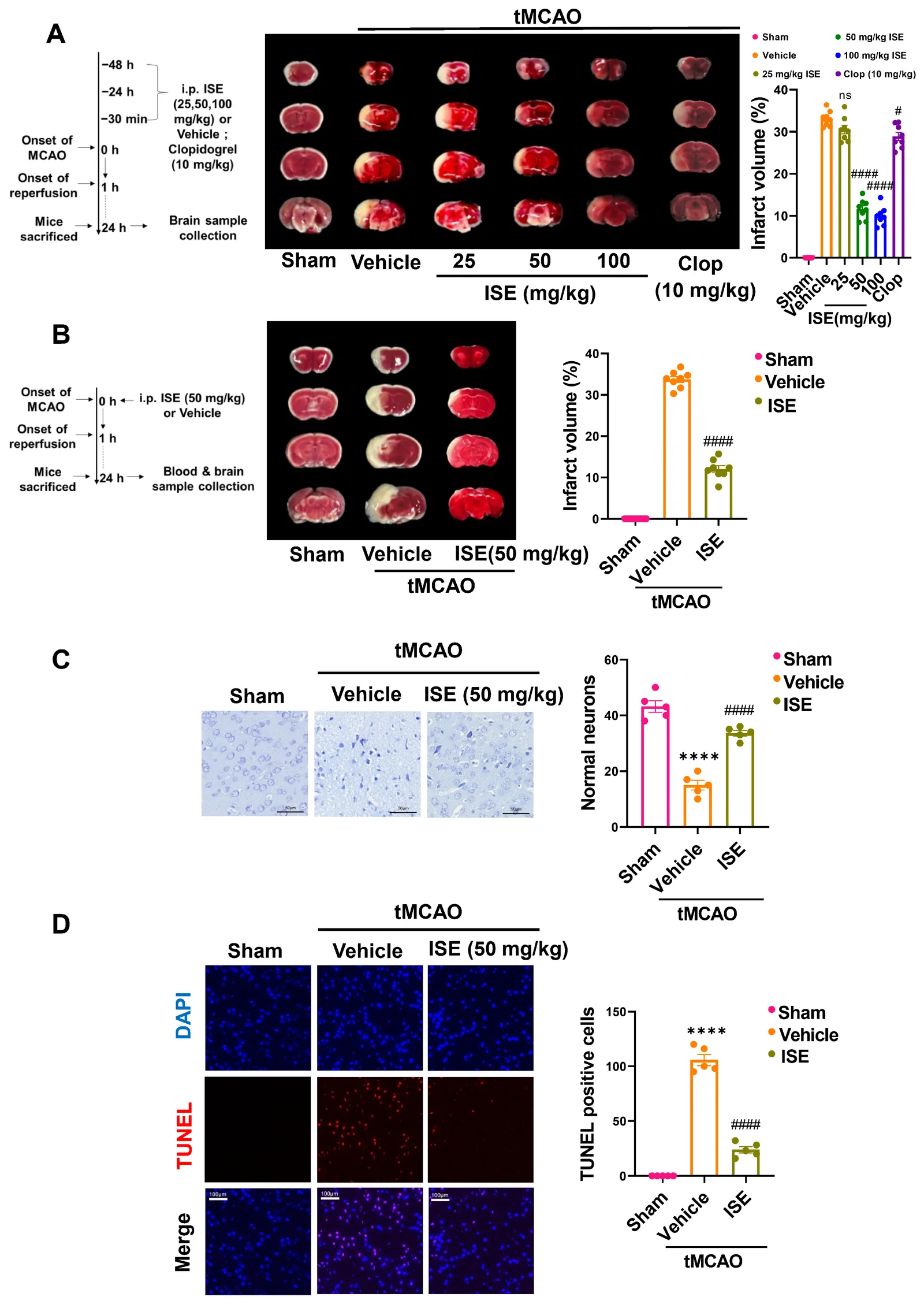
ISE attenuated cerebral infarct volume, neuronal injury, and apoptosis in tMCAO mice. (**A**,**B**) Quantitative analysis and representative images of TTC-stained brain sections. Red staining indicates normal brain tissue, whereas the infarct area appears white. Sham mice underwent surgery without ischemia. (**A**) tMCAO mice were pretreated with multiple injections of vehicle or ISE (25, 50, and 100 mg/kg) or clopidogrel (10 mg/kg). *n* = 8 mice per group. (**B**) tMCAO mice received a single injection of vehicle or ISE (50 mg/kg) at the onset of ischemia. *n* = 8 mice per group. (**C**) Nissl staining of the ischemic penumbra of the Sham and tMCAO mice therapeutically treated with vehicle or ISE (Left). Quantitative analysis of the number of intact neurons (Right). Scale bar = 50 µm. *n* = 5 mice per group. (**D**) Representative TUNEL staining images of the ischemic penumbra of the Sham and tMCAO mice therapeutically treated with vehicle or ISE (Left). Quantitative analysis of the number of TUNEL-positive cells (Right). Scale bar = 100 µm. *n* = 5 mice per group. ns: *p* > 0.05, **** *p* < 0.0001 vs. the sham group; ^#^ *p* < 0.05, ^####^ *p* < 0.0001 vs. the vehicle + tMCAO group.

We first assessed the effect of ISE on ischemic brain damage by measuring cerebral infarct volume using TTC staining. As shown in Figure 1A, mice were pretreated with multiple injections of vehicle or different doses of ISE (25, 50, and 100 mg/kg) intraperitoneally at 48 h, 24 h, and 30 min before ischemia. Clopidogrel (10 mg/kg), the cornerstone antiplatelet agent for ischemic stroke, was used as a positive control. This ISE preventive regimen reduced the relative infarct volumes to 30.5 ± 1.0% (25 mg/kg), 11.6 ± 0.8% (50 mg/kg), and 10.0 ± 0.8% (100 mg/kg) in a dose-dependent manner, compared to 33.1 ± 0.7% in the vehicle-treated tMCAO group. Clopidogrel pretreatment also significantly reduced the relative infarct volume compared to the vehicle-treated tMCAO group. Furthermore, a single therapeutic dose of ISE (50 mg/kg, administered intraperitoneally at ischemia onset) also significantly reduced the relative infarct volume to 12.0 ± 0.8%, versus 33.7 ± 0.7% in the vehicle-treated tMCAO group (Figure 1B).

Then, we evaluated neuronal injury by Nissl staining. As shown in Figure 1C, vehicle-treated tMCAO mice showed a significant reduction in intact neurons, characterized by markedly depleted or dispersed Nissl bodies compared with the sham-operated (sham) group. However, therapeutic administration of ISE (50 mg/kg) attenuated this neuronal injury, increasing the number of intact neurons and such that the overall cytoarchitecture closely resembled that of sham controls.

Neuronal apoptosis was further assessed by TUNEL staining. As shown in Figure 1D, therapeutic ISE (50 mg/kg) treatment significantly reduced the number of apoptotic cells (TUNEL-positive) in the ischemic penumbra compared to the vehicle-treated tMCAO mice.

To evaluate the effect of ISE on post-stroke neurological function, neurological deficits were assessed at 24 h after ischemia using a 4-tiered scoring system. As summarized in Table 1 and Table 2, the sham group showed no neurological impairment, whereas vehicle-treated tMCAO mice exhibited severe neurological impairment. Notably, both preventive (50 and 100 mg/kg) (Table 1) and therapeutic (50 mg/kg) (Table 2) administration of ISE significantly improved the neurological outcome of the tMCAO mice, compared to the vehicle-treated group. In contrast, clopidogrel pretreatment (10 mg/kg) did not significantly ameliorate the neurological deficit scores under the same experimental conditions (Table 1), although it significantly reduced the cerebral infarct volume (Figure 1A).

Taken together, these histopathological and neurological function analyses suggested that ISE may be a promising neuroprotective candidate against ischemic stroke. Based on the results, the single dose of 50 mg/kg ISE at ischemia onset (therapeutic regimen) was chosen for subsequent experiments to explore its underlying mechanism.

### 2.2. ISE Attenuated Platelet Hyperactivity and Circulating Pro-Inflammatory Mediators in tMCAO Mice

**Figure 2:**
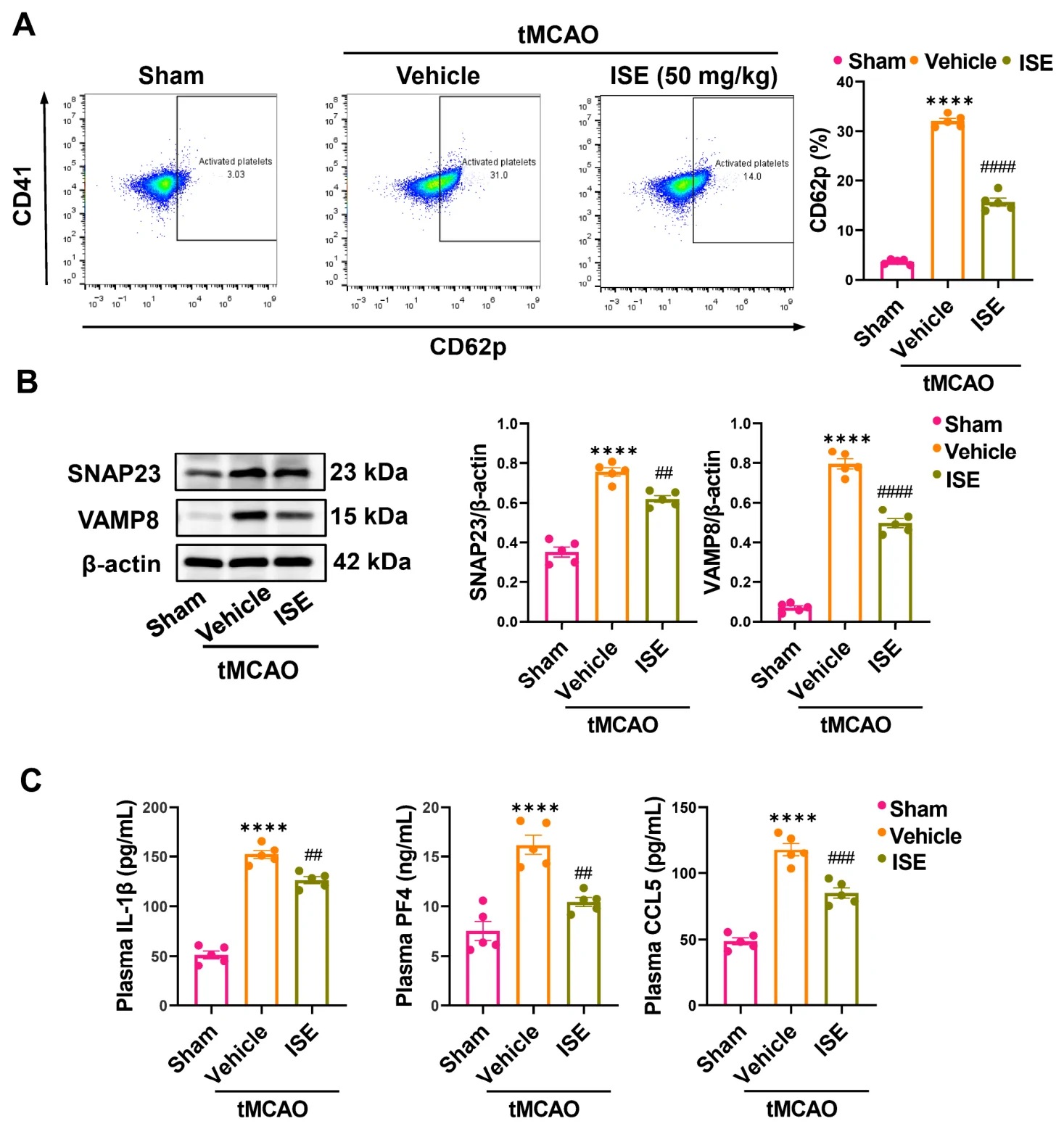
ISE attenuated tMCAO-induced platelet hyperactivity and circulating platelet-derived inflammatory factors. (**A**) Flow cytometric analysis of CD41 and CD62p expression on washed platelets isolated from the sham, vehicle-treated tMCAO, and ISE-treated tMCAO mice. (**B**) Western blot analysis of SNAP23 and VAMP8 protein expression. (**C**) ELISA analysis of plasma PF4, IL-1β, and CCL5. *n* = 5 mice per group. **** *p* < 0.0001 vs. the sham group; ^##^ *p* < 0.01, ^###^ *p* < 0.001, ^####^ *p* < 0.0001 vs. the vehicle + tMCAO group.

Platelets act as fine-tuned regulators coordinating the tight interplay between thrombosis and inflammation, which renders them a promising therapeutic target for ischemic stroke [20,21]. We have demonstrated previously that ISE specifically suppresses ADP-induced platelet aggregation, secretion and activation in vitro [19]. Here, to determine whether ISE affects I/R-induced platelet activation in tMCAO mice, we performed flow cytometry to measure the surface expression of P-selectin (CD62p), an important platelet activation marker. As shown in Figure 2A, the percentage of CD62p-positive platelets increased to 32.0 ± 0.5% in vehicle-treated tMCAO mice, compared to 3.7 ± 0.2% in the sham group. Yet, therapeutic administration of ISE significantly attenuated this elevated platelet activation, reducing the percentage to 15.7 ± 0.8%.

Building on our prior finding that ISE potently inhibits platelet secretion at low micromolar concentrations (IC_50_ ≈ 7.9 μM for ADP-induced ATP release, markedly lower than that for aggregation [19]), we next examined whether ISE modulates the expression of platelet secretion-related membrane proteins in tMCAO mice. As shown in Figure 2B, therapeutic ISE treatment significantly suppressed the upregulated protein levels of platelet granule secretion proteins SNAP23 (soluble N-ethylmaleimide-sensitive factor attachment protein 23) and VAMP8 (vesicle-associated membrane protein 8), two critical SNARE components governing platelet granule exocytosis [22,23], in platelets from tMCAO mice. Moreover, as presented in Figure 2C, therapeutic treatment with ISE significantly alleviated the elevated plasma protein levels of PF4, IL-1β, and CCL5 in tMCAO mice. Collectively, these data indicate that therapeutic administration of ISE (50 mg/kg) suppressed platelet activation and the consequent release of platelet-derived pro-inflammatory mediators (PF4, IL-1β, and CCL5) into the circulation.

### 2.3. ISE Attenuated Neuroinflammation in tMCAO Mice

**Figure 3:**
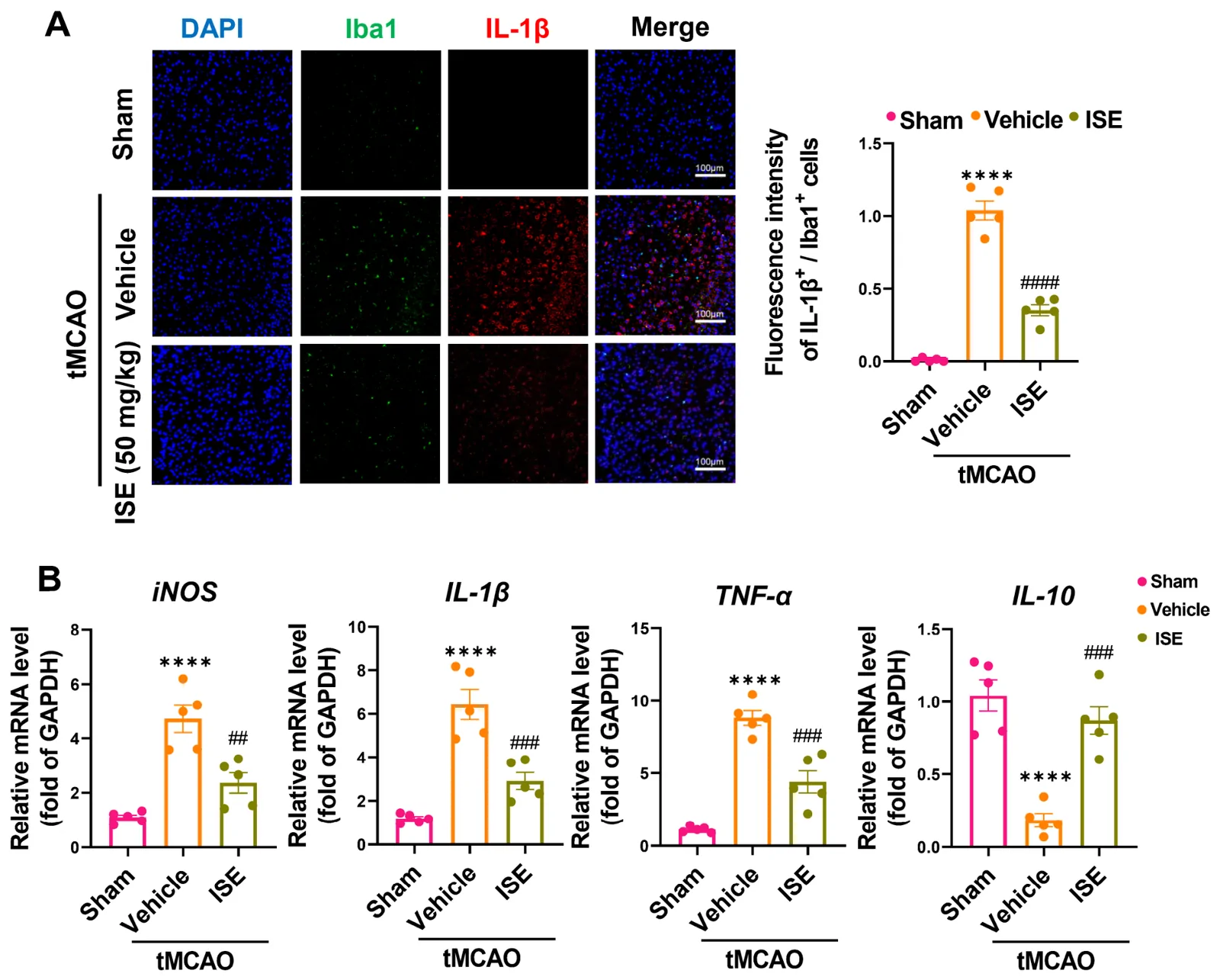
ISE attenuated neuroinflammation in tMCAO mice. (**A**) Immunofluorescence staining of Iba1 (green) and IL-1β (red) in the ischemic penumbra. Scale bar = 100 μm. (**B**) qRT-PCR analysis of the cytokine mRNA levels (*iNOS*, *IL-1β*, *TNF-α* and *IL-10*) in the ischemic penumbra. *n* = 5 mice per group. **** *p* < 0.0001 vs. the sham group; ^##^ *p* < 0.01, ^###^ *p* < 0.001, ^####^ *p* < 0.0001 vs. the vehicle + tMCAO group.

Neuroinflammation within the central nervous system is a key mechanism of post-stroke injury and recovery, due to its critical role in blood–brain barrier disruption, cerebral edema, and apoptosis [24,25]. Microglia act as important mediators of neuroinflammation and, based on their phenotype and activity, as reliable indicators for its detection [26,27]. As shown in Figure 3A, the protein level of IL-1β was significantly upregulated in Iba1 (a commonly used microglial marker) positive cells within the ischemic penumbra compared to the sham group, whereas therapeutic ISE treatment significantly reduced this upregulation. Moreover, therapeutic ISE treatment significantly mitigated the mRNA levels of pro-inflammatory cytokines (*iNOS*, *IL-1β*, and *TNF-α*) while elevating anti-inflammatory cytokine *IL-10* in the lesioned hemisphere (Figure 3B). Taken together, these data suggest that therapeutic ISE (50 mg/kg) treatment may attenuate microglial inflammatory activation and associated neuroinflammation in the post-ischemic brain.

### 2.4. ISE Attenuated Microglial Inflammatory Activation via the LRP1/IκB/NF-κB Signaling Pathway In Vitro

Low-density lipoprotein receptor-related protein 1 (LRP1), an efferocytosis receptor widely expressed on microglia, interacts with membrane receptors and intracellular adaptors to regulate the levels of numerous extracellular molecules [28]. It has been found that microglia from microglial LRP1 conditional knockout mice exhibit a pro-inflammatory phenotype, indicating a critical role of LRP1 in microglial activation [29]. Based on the attenuation of neuroinflammation observed in vivo (Figure 3), and given that microglia are key effectors of post-ischemic inflammation, we next investigated whether LRP1 contributes to the ISE-induced suppression of microglia-associated neuroinflammation in vitro. Primary microglia isolated from neonatal mice were used in the following experiments. We first examined whether ISE affects primary microglia viability using the CCK-8 assay. As shown in Figure S1, ISE exerted no significant effect on primary microglia viability at concentrations of 12.5, 25, 50, 100, or 200 μM.

**Figure 4:**
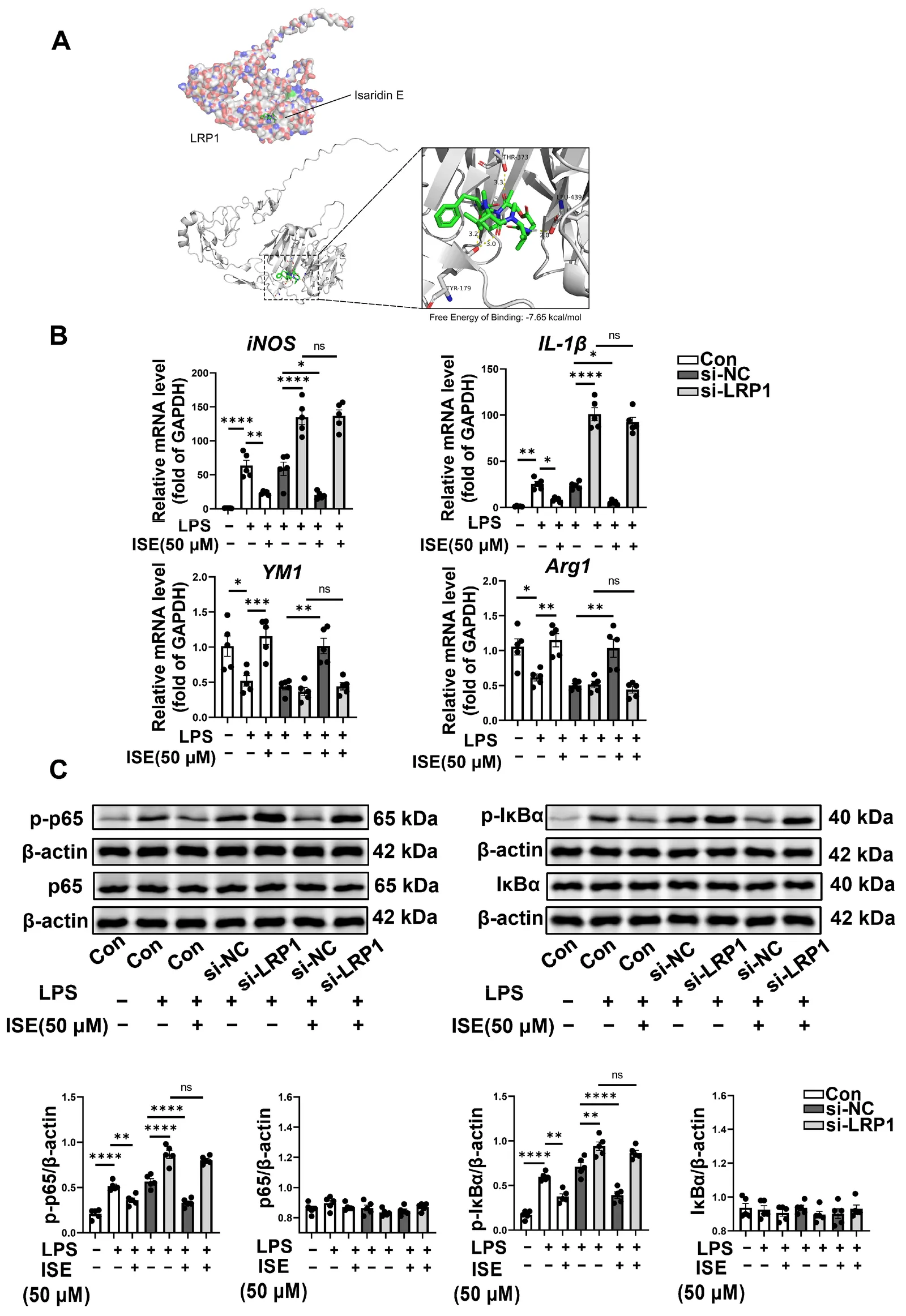
ISE attenuated microglial inflammatory activation through the LRP1/IκB/NF-κB pathway in vitro. (**A**) Molecular docking of ISE with LRP1. LRP1 is shown in both mesh and cartoon representations, and ISE is depicted in stick representation. (**B**,**C**) Primary microglia were transfected with si-NC (negative control siRNA) or si-LRP1 (siRNA targeting LRP1) for 48 h, then pretreated with ISE (50 μM) for 30 min before exposure to LPS (100 ng/mL). LPS stimulation for 30 min was used for Western blot analysis because phosphorylation of IκBα/NF-κB peaks early after LPS stimulation, whereas a 12 h stimulation was used for qRT-PCR analysis because LPS-induced inflammatory gene transcription peaks at this time point. The mRNA levels of *iNOS*, *IL-1β*, *YM1* and *Arg1* were determined by qRT-PCR (**B**). And the protein expression of p-IκBα, IκBα, p-p65, p65 and β-actin was analyzed by Western blot (**C**). *n* = 5 independent experiments. ns: *p* > 0.05, * *p* < 0.05, ** *p* < 0.01, *** *p* < 0.001, **** *p* < 0.0001.

As shown in Figure 4A, molecular docking analysis was performed. The calculated binding energy between ISE and LRP1 was −7.65 kcal/mol, forming three hydrogen bonds with key amino acid residues of LRP1, including THR-373, LEU-439 and TYR-179, supporting a stable theoretical interaction. To validate this docking prediction, primary microglia were transfected with small interfering RNA targeting LRP1 (si-LRP1) to decrease the corresponding protein expression levels. Endogenous LRP1 protein expression was remarkably reduced to 37% of control by transfection with 75 nM si-LRP1 for 48 h (Figure S2). We then established an in vitro inflammatory model by stimulating primary microglia with LPS (100 ng/mL). As shown in Figure 4B, LPS induced microglial inflammatory activation, with markedly increased *iNOS* and *IL-1β* levels and decreased *Arg1* and *YM1* levels, all of which were significantly reversed by ISE. Consistent with these data, ISE at 50 μM abolished LPS-induced microglial inflammatory activation in si-NC-transfected cells. However, silencing LRP1 significantly aggravated LPS-induced microglial inflammatory activation, and ISE had only a minimal inhibitory effect on this activation in LRP1-deficient microglia. These results suggested that ISE inhibited microglial inflammatory activation predominantly via the LRP1 signaling pathway.

Previous studies have identified LRP1 as a negative regulator of NF-κB activation in microglial activation [29,30,31]. We further investigated whether ISE modulates microglial inflammatory activation via the LRP1/IκB/NF-κB axis in vitro. As presented in Figure 4C, upon LPS stimulation, the phosphorylation of IκBα and NF-κB was significantly upregulated, whereas ISE (50 μM) significantly suppressed the enhanced phosphorylation of IκBα and NF-κB without altering total protein expression. However, these changes were absent in si-LRP1-transfected microglia in which ISE had no significant effects on LPS-induced IκBα and NF-κB phosphorylation or total protein expression. Taken together, these results suggested that LRP1 may serve as the target mediating the effects of ISE on microglia-associated neuroinflammation following cerebral I/R injury; these LRP1-dependent effects were observed in vitro and warrant in vivo validation.

## 3. Discussion

Marine-derived natural products are promising candidates for stroke therapy owing to their structural diversity and multi-target pharmacological potential, including antiplatelet and anti-inflammatory activities [18]. Herein, we found for the first time that the marine-derived cyclodepsipeptide ISE exerts neuroprotective effects against ischemic stroke in tMCAO mice by concurrently suppressing platelet hyperactivity and microglia-associated neuroinflammation. This finding extends the pharmacological profile of ISE beyond its established antithrombotic activity [19], revealing a previously unrecognized capacity to modulate neuroinflammatory responses in the post-ischemic brain. Mechanistically, our data suggest a dual-action model in which ISE suppresses platelet activation and secretion, thereby curtailing the release of pro-inflammatory mediators into the circulation, while concurrently engaging the LRP1/IκB/NF-κB axis to suppress microglial inflammatory activation. These results suggest that ISE represents a structurally unique marine metabolite with potential for further development as a multi-mechanism therapeutic agent against ischemic stroke.

Antiplatelet therapy is the cornerstone of antithrombotic treatment in the management of ischemic stroke to prevent ischemic stroke recurrence, especially in cases caused by large-artery extracranial atherosclerosis or intracranial small vessel disease [32]. Although the efficacy of single or dual antiplatelet therapy (aspirin or aspirin plus clopidogrel, respectively) in ischemic stroke is well studied, the increased risk of bleeding and potential risk of hemorrhagic transformation remain major concerns [15]. However, whether these clinically used antiplatelet therapies protect against brain injury in patients with ischemic stroke remains unclear, despite experimental studies showing the effects of clopidogrel on neuroinflammation [33,34]. Together, these considerations highlight the urgent need for novel and multifunctional antiplatelet agents without increased bleeding risk. As reported in our previous study [19], ISE at 50 or 100 mg/kg exerted potent anti-thrombotic effects comparable to those of clopidogrel at 10 mg/kg in the FeCl_3_-induced carotid artery thrombosis mouse model, yet with a lower bleeding risk. The preferential suppression of platelet secretion over aggregation may account for the favorable bleeding safety profile of ISE. Our previous work demonstrated that ISE inhibits ADP-induced platelet ATP release with an IC_50_ of approximately 7.9 μM, a potency substantially greater than that for aggregation (IC_50_ approximately 26.1 μM) [19], suggesting a selective action on the secretory machinery. The present finding that ISE reduces the expression of SNAP23 and VAMP8, core SNARE components governing platelet granule exocytosis, provides a molecular rationale for this preferential inhibition. Unlike conventional P2Y_12_ antagonists such as clopidogrel, which interrupt the ADP–P2Y_12_–Gi–PI3Kβ signaling cascade to suppress integrin αIIbβ3 activation and fibrinogen-mediated platelet aggregation, thereby inevitably compromising primary hemostasis and predisposing to bleeding [35,36], ISE may preferentially target the SNARE-related secretory machinery. By dampening the release of pro-thrombotic and pro-inflammatory mediators while preserving integrin-mediated aggregation induced by collagen or thrombin as we previously reported [19], ISE may dissociate pathological thrombotic propagation from physiological hemostatic plug formation, offering a mechanistic basis for its antithrombotic efficacy without prolonging bleeding time. Collectively, these properties identify ISE as a promising antiplatelet candidate for both prevention and treatment of ischemic stroke. However, whether ISE affects hemorrhagic transformation in ischemic stroke mice remains to be determined. Moreover, the in vitro experiments in the present study employed ISE at 50 μM; whether this concentration is achievable in the plasma or the ischemic brain microenvironment after intraperitoneal administration of ISE (50 mg/kg) remains to be determined by pharmacokinetic studies. In addition, the long-term efficacy and safety of ISE during post-ischemic recovery warrant further investigation.

While platelet activation initiates the inflammatory cascade in the acute phase of stroke, microglial activation represents the central executioner of neuroinflammation that drives secondary brain injury [37]. LRP1 serves as a pivotal gatekeeper that maintains microglial homeostasis, and its loss exacerbates pro-inflammatory responses [29,31]. Herein, we provide the first evidence that ISE attenuates microglial pro-inflammatory activation through modulation of the LRP1/IκB/NF-κB axis. Our in vitro findings demonstrated that ISE suppresses LPS-induced upregulation of pro-inflammatory markers in an LRP1-dependent manner, an effect largely abolished upon LRP1 silencing. Molecular docking further indicated a favorable interaction between ISE and LRP1, although whether ISE functions as a direct small-molecule agonist of LRP1 remains to be validated using a surface plasmon resonance (SPR) assay. These results suggest that microglial LRP1 may serve as a previously unrecognized cellular mediator linking the anti-neuroinflammatory activity of ISE to the regulation of microglial pro-inflammatory phenotype. However, as these LRP1-dependent effects were observed in vitro, future studies employing microglial LRP1 conditional knockout mice are needed to confirm this role in vivo. In parallel with this central action, ISE concurrently suppresses platelet hyperactivity and attenuates the associated release of pro-inflammatory mediators into the circulation. Given that platelets, through synergistic interactions with neutrophils and other immune cells, are increasingly recognized as key effector cells in the intricate pathological loop underlying acute neuroinflammatory diseases such as stroke and TBI [38], whether the effects of ISE against platelet hyperactivity and microglia-associated neuroinflammation in tMCAO mice have a direct causal link remains to be clarified in future studies.

Notably, ISE has recently been shown to ameliorate LPS-induced vascular endothelial inflammation via downregulation of the TLR4/NF-κB signaling pathway [39], suggesting that endothelial protection may represent an additional mechanism contributing to its neuroprotective efficacy. Together, these findings support a multi-cellular protective model of ISE encompassing platelets, endothelium, and microglia within the post-ischemic neurovascular unit [40]. Given this broad mechanistic profile coupled with a favorable bleeding safety profile, ISE may represent remarkable translational potential for ischemic stroke therapy, suggesting its promise as an adjunctive therapy in future studies.

## 4. Materials and Methods

### 4.1. Preparation and Administration of ISE

**Figure 5:**
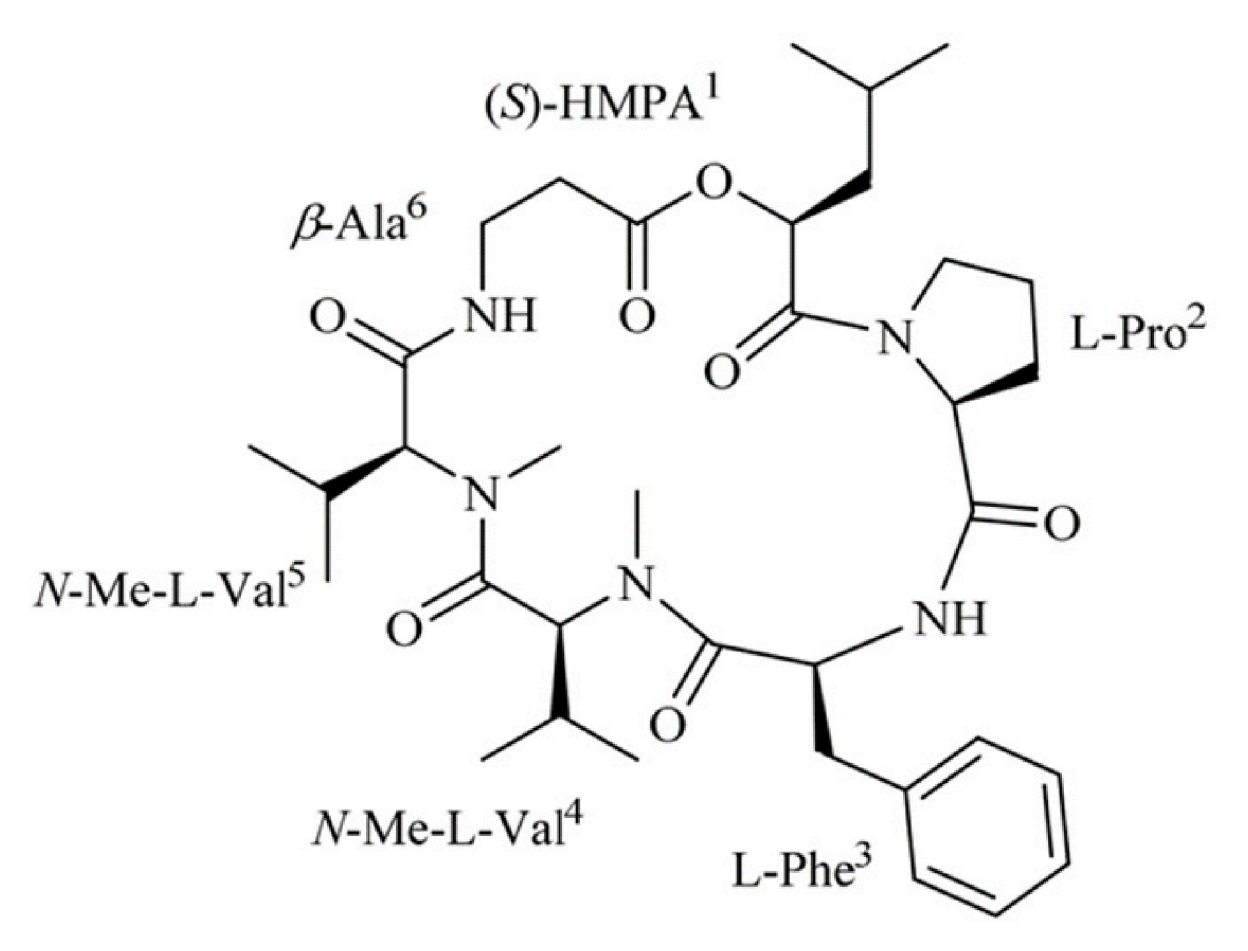
The chemical structure of ISE.

ISE is a fungal cyclodepsipeptide isolated from the marine-derived fungus *Amphichorda felina* (syn. *Beauveria felina*) SYSU-MS7908. ISE was isolated and purified as previously described [19]. ISE was obtained as a colorless crystal with a molecular formula of C_35_H_54_O_7_N_5_, and its identity was confirmed by ^1^H and ^13^C NMR and HR-ESIMS. The chemical structure of ISE is shown in Figure 5, as previously reported, and the purity of ISE > 98.5% [19]. For intraperitoneal administration, ISE was dissolved in a saline solution containing 10% Tween 80 and 15% propylene glycol (used as a vehicle solution) at different doses of ISE (25, 50, and 100 mg/kg). The same vehicle without ISE served as the control. For the prophylactic regimen, ISE (25, 50, or 100 mg/kg, i.p.) was administered at 48 h, 24 h, and 30 min before ischemia. For the therapeutic regimen, ISE (50 mg/kg, i.p.) was administered once at the onset of ischemia (immediately after MCA occlusion). As a positive control, clopidogrel (10 mg/kg, i.p.) was administered at 48 h, 24 h, and 30 min before ischemia.

For in vitro experiments, ISE was prepared in dimethyl sulfoxide (DMSO) and stored at 4 °C until use, with the final DMSO concentration in culture medium less than 0.1% [19].

### 4.2. Animals

Male C57BL/6 mice (8–10 w, 20–22 g) were obtained from the Laboratory Animal Center of Sun Yat-sen University. All animal procedures were approved by the Sun Yat-sen University Animal Care and Use Committee (Approval No: SYSU-IACUC-2024-B0222) and strictly followed the “Guide for the Care and Use of Laboratory Animals” issued by the Ministry of Science and Technology of China. All experimental procedures were designed to use the minimum number of animals necessary while minimizing their pain and distress. Animals were randomly assigned to the experimental groups before treatment by computer-generated randomization schedules.

### 4.3. Transient Middle Cerebral Artery Occlusion (tMCAO) Mouse Model

Transient focal cerebral I/R injury was induced by tMCAO as previously described [41,42]. Briefly, after the mice were anesthetized with sodium pentobarbital (Merck, New Zealand, USA; 60 mg/kg, i.p.), we exposed the left common carotid artery (CCA), external carotid artery (ECA), and internal carotid artery (ICA). A silicone-coated suture (Cinontech, Beijing, China) was then gently inserted into the ECA and advanced through its lumen into the ICA until it blocked the origin of the middle cerebral artery (MCA). Occlusion was maintained for 60 min, followed by withdrawal of the filament to allow reperfusion. Regional cerebral blood flow (rCBF) in the MCA territory was continuously monitored by laser-Doppler flowmetry (RWD Biotechnology, Shenzhen, China) before ischemia, during MCA occlusion, and after filament withdrawal. A stable pre-ischemic perfusion value was recorded as baseline. Successful MCA occlusion was defined as a reduction in rCBF of ≥75% from baseline, and successful reperfusion was defined as recovery of rCBF to ≥70% of basal cerebral blood flow within 10 min after filament withdrawal. Animals that failed to meet these predefined criteria were excluded from subsequent analyses. Sham-operated mice underwent the same procedure without filament insertion. After 24 h of ischemia, the brains and the whole blood were collected and subjected to different procedures based on the type of analysis.

### 4.4. Neurological Deficit Scoring

Neurological deficits were scored at 24 h after ischemia according to the Longa neurological deficit scale (0–4) in a double-blind manner. The scoring criteria were as follows: 0, no neurological deficits; 1, failure to fully extend the contralateral forelimb; 2, spontaneous circling to the contralateral side; 3, leaning/falling to the contralateral side; 4, no spontaneous walking or loss of consciousness [43].

### 4.5. TTC Staining

At 24 h after ischemia, the mice were euthanized, and the brains were removed and immediately frozen at −20 °C for 30 min. Then the frozen brains were sectioned into 2 mm coronal slices. The brain slices were stained with 2% 2,3,5-triphenyltetrazolium chloride (TTC, T8877, Sigma-Aldrich, St. Louis, MO, USA) at 37 °C for 10 min and fixed in 4% paraformaldehyde as previously described [44]. Images of the stained slices were captured using a scanner and analyzed with ImageJ 2.1.0 software to measure the infarct volume. The infarct volume was calculated using the following formula: Corrected infarct volume (%) = [contralateral hemisphere volume − (ipsilateral hemisphere-infarct volume)]/contralateral hemisphere volume × 100%.

### 4.6. Nissl Staining

Nissl staining was performed using Toluidine Blue O (T8240, Solarbio, Beijing, China) on brain sections to assess histological injury. Briefly, paraffin-embedded sections were dewaxed by heating at 60 °C, immersed in xylene, and then rehydrated through a graded ethanol series, followed by a rinse in double-distilled water. Subsequently, the sections were stained with Toluidine Blue O and differentiated in acetic acid solution. After being mounted, the stained sections were imaged using an Olympus BX63 microscope (Olympus, Tokyo, Japan), and images were then analyzed with ImageJ 2.1.0 software.

### 4.7. TUNEL Staining

TUNEL staining was performed using a TUNEL Apoptosis Assay kit (T2195, Solarbio, Beijing, China) according to the manufacturer’s instructions. After being dewaxed and rehydrated, brain sections were subjected to antigen retrieval in a Proteinase K working solution (20 μg/mL) at 37 °C for 20 min. The sections were rinsed with PBS and then incubated with TUNEL reaction mixture at 37 °C in the dark for 1 h. Finally, the stained sections were mounted with an anti-fluorescence quenching agent and coverslips. Images were acquired using an Olympus BX63 microscope (Olympus, Tokyo, Japan) and analyzed with ImageJ 2.1.0 software.

### 4.8. Preparation of Mouse Platelets

Washed platelets were prepared as we previously described [19]. In brief, whole blood was centrifuged at 300× *g* for 2 min at room temperature to obtain platelet-rich plasma (PRP). Platelets were pelleted by further centrifugation of PRP at 700× *g* for 2 min and resuspended in Tyrode’s buffer (138 mM NaCl, 2.7 mM KCl, 1 mM MgCl_2_, 3 mM Na_2_HPO_4_, 10 mM HEPES, 5 mM glucose, pH 7.4) with 0.02 U/mL apyrase for use.

### 4.9. Flow Cytometry Analysis

Platelet samples from sham-operated, vehicle-treated and ISE-treated mice were stained with a PE-conjugated anti-CD62p antibody (148306, BioLegend, San Diego, CA, USA) and FITC-conjugated anti-CD41 antibody (133904, BioLegend, San Diego, CA, USA) for 20 min in the dark at room temperature. Then the stained platelets were fixed in 1% paraformaldehyde solution and analyzed on a CytoFLEX flow cytometer (Beckman Coulter, Brea, CA, USA) as we described previously [19].

### 4.10. Western Blotting Assay

The Western blot experiment was carried out as we described previously [19]. Protein samples (30 µg) were separated on 8–12% SDS-polyacrylamide gels and transferred onto polyvinylidene difluoride (PVDF) membranes. The membranes were blocked and then incubated with primary antibodies overnight at 4 °C, followed by incubation with HRP-conjugated secondary antibodies. The immunoreactive bands were detected using an enhanced chemiluminescence substrate with the ChemiDoc XRS+ system (Bio-Rad, Hercules, CA, USA) and quantified with ImageJ 2.1.0 software.

The following primary antibodies were used: anti-VAMP8 (ab76021, Abcam, 1:1000), anti-SNAP23 (ab133703, Abcam, 1:1000), anti-phospho-NF-κB p65 (Ser536) (#3033S, CST, 1:1000), anti-NF-κB p65 (#8242S, CST, 1:1000), anti-phospho-IκBα (Ser32/36) (#9246, CST, 1:1000), anti-IκBα (#9247, CST, 1:1000), anti-LRP1 (ab92544, Abcam, 1:1000) and anti-β-actin (66009-1, Proteintech, 1:5000).

### 4.11. Enzyme-Linked Immunosorbent Assay

Levels of IL-1β (E-EL-M0037, Elabscience, Wuhan, China), PF4 (ml037257, Mibio, Shanghai, China) and CCL5 (E-EL-M0009, Elabscience, Wuhan, China) in mouse plasma were measured using enzyme-linked immunosorbent assay (ELISA) kits according to the manufacturers’ protocols. Briefly, after blocking with 1% casein, the plasma samples were added to the antibody-coated 96-well plate, followed by incubation with horseradish peroxidase-labeled detection antibody. The signal was developed using 3,3′,5,5′-tetramethylbenzidine substrate and absorbance was measured at 450 nm using a Sunrise^TM^ ELISA plate reader (Tecan, Zurich, Switzerland). The standard curve was obtained by plotting the average optical density (Y-axis) of standard samples against their known concentration (X-axis). Plasma sample concentrations were calculated on the standard curve.

### 4.12. Quantitative Real-Time Polymerase Chain Reaction (qRT-PCR)

Quantitative real-time PCR was performed as we previously described [39]. Briefly, total RNA from the ischemic penumbra was extracted using TRIzol reagent (Invitrogen, Carlsbad, CA, USA). Then 1 µg of total RNA was reverse-transcribed into complementary DNA for the SYBR Green-based real-time PCR on a CFX96 Touch Real-Time PCR Detection System (Bio-Rad, Hercules, CA, USA). The reactions were run under the following cycle conditions: initial denaturation at 95 °C for 10 min, followed by 40 cycles of 95 °C for 10 s and 60 °C for 1 min. The melting curve analysis was performed immediately after amplification to verify the specificity of the PCR products, consisting of 50 cycles of 10 s holds with the temperature increasing 0.5 °C/cycle. The relative mRNA of each target gene was calculated using the 2^−ΔΔCt^ method, using GAPDH mRNA as an internal control.

The primer sequences used for qRT-PCR are listed in Table 3.

### 4.13. Immunofluorescence Analysis

Immunofluorescence staining was performed as we previously described [39]. The dewaxed and rehydrated sections were exposed to the primary antibodies at 4 °C overnight, and then reacted with fluorophore-conjugated secondary antibodies (Cy3-labeled anti-mouse IgG, ab97035, Abcam; FITC-labeled anti-rabbit IgG, ab6717, Abcam) for 1 h at room temperature in the dark. After being mounted in a DAPI-containing anti-fade medium, the sections were imaged using an Olympus BX63 microscope (Olympus, Tokyo, Japan) and fluorescence images were analyzed with ImageJ 2.1.0 software.

The following primary antibodies were used: anti-Iba1 (019-19741, Wako, 1:500), anti-IL-1β (ab156791, Abcam, 1:500).

### 4.14. Molecular Docking

The AlphaFold-predicted human LRP1 structure (AF-Q6PJ72-F1, model version 6; UniProt accession Q6PJ72) was used for molecular docking, with low-confidence terminal and non-structured regions excluded. The structure of ISE was obtained from the PubChem database. Receptor and ligand structures were prepared using Open Babel (v3.1.1) and MGLTools (v1.5.7).

Molecular docking was performed using AutoDock 4.2.6 with the Lamarckian Genetic Algorithm. The docking grid was centered at (−0.667, −0.771, 10.940 Å), with 126 × 88 × 126 grid points and a spacing of 0.375 Å. A total of 100 independent docking runs were performed. The representative pose was selected from the top-ranked conformations based on predicted binding affinity and the overall ligand–receptor interaction pattern. The selected pose corresponded to run 21, with an estimated binding free energy of −7.65 kcal/mol and a predicted inhibition constant of 2.48 μM. Docking interactions were visualized using PyMOL (v3.1.6.1).

### 4.15. Primary Microglia Culture

Primary microglia were isolated from postnatal day 1–2 (P1–P2) C57BL/6 mouse neonates, as previously described [45]. Briefly, after removing the meninges, brains were dissociated and trypsinized. The cell suspension was passed through a 70 μm cell strainer (Corning, Corning, NY, USA), and the resulting mixed glial cells were cultured in DMEM/F12 (Gibco, Grand Island, NY, USA) supplemented with 10% fetal bovine serum (Gibco, Grand Island, NY, USA) and 1% penicillin-streptomycin (Gibco, Grand Island, NY, USA) in a humidified incubator at 37 °C with 5% CO_2_. Microglia were mechanically isolated from the mixed glial cultures by shaking on a rotary shaker at 7.5× *g* for 4 h at 37 °C. The detached microglia were plated onto new culture dishes and maintained at 37 °C in a humidified incubator with 5% CO_2_ until use. Microglial purity (>95%) was confirmed by immunofluorescence staining of the microglial marker Iba1.

Primary microglia were transfected with si-NC or si-LRP1 and pretreated with vehicle or ISE (50 μM) for 30 min at 37 °C before exposure to LPS (100 ng/mL), a pretreatment duration based on our previous study demonstrating that 30 min ISE pretreatment effectively suppresses agonist-induced activation and PI3K/Akt signaling [19]. LPS stimulation was applied for 30 min for Western blot analysis, as phosphorylation of IκBα/NF-κB occurs rapidly and peaks early after LPS stimulation, whereas a 12 h stimulation was used for qRT-PCR analysis, as LPS-induced inflammatory gene transcription peaks at approximately 12 h.

### 4.16. CCK-8 Assay

Primary microglia were treated with vehicle or ISE (12.5, 25, 50, 100, 200 μM) for 24 h. Cell viability was assessed using the Cell Counting Kit-8 (CK04, Solarbio, Beijing, China) assay according to the manufacturer’s instructions. Cell viability was measured at 450 nm using a microplate reader (Tecan, Zurich, Switzerland).

### 4.17. siRNA Transfection

si-NC (negative control siRNA) and si-LRP1 (siRNA targeting LRP1), both synthesized by Ribo (Guangzhou, China), were transiently transfected into cells using Lipofectamine 3000 reagent (Invitrogen, Carlsbad, CA, USA) according to the manufacturer’s protocols. A mixture of siRNA and P3000^TM^ reagent was prepared in Opti-MEM^TM^ medium. This siRNA mixture was then added to the diluted Lipofectamine^TM^ 3000 reagent, incubated at room temperature for 10–15 min to form complexes, and subsequently added to cells for transfection.

The target sequence used for LRP1 siRNA: 5′-GGAGUCACUUACAUCAAUAUU-3′.

### 4.18. Statistical Analysis

Data are presented as mean ± SEM. Statistical analyses were performed using GraphPad Prism (Version 11, GraphPad Software, La Jolla, CA, USA). Two-group comparisons were assessed using an unpaired two-tailed Student’s *t*-test. Multiple group comparisons were assessed using one-way ANOVA followed by Tukey’s multiple comparisons post hoc testing. For non-normally distributed data, the Kruskal–Wallis test was used followed by Dunn’s multiple comparisons. The value of *p* < 0.05 was considered statistically significant.

## 5. Conclusions

In summary, the present study suggests that ISE protects mice against ischemic stroke by concurrently suppressing platelet hyperactivity and attenuating microglial inflammatory activation. In vitro mechanistic studies indicate that the LRP1/IκB/NF-κB axis serves as a key mediator of the anti-neuroinflammatory effects of ISE. Coupled with its favorable bleeding safety profile, these findings highlight the potential of ISE as a multi-target marine-derived compound for translation into ischemic stroke therapy.

## Figures and Tables

**Table 1:** Preventive effects of ISE on neurological deficit scores in tMCAO mice.

Group	Sham	Vehicle	ISE (25 mg/kg)	ISE (50 mg/kg)	ISE (100 mg/kg)	Clopidogrel (10 mg/kg)
#1	0	2	1	2	1	2
#2	0	3	2	1	1	3
#3	0	3	3	1	2	2
#4	0	3	3	2	2	3
#5	0	2	2	1	2	2
#6	0	4	2	1	1	2
#7	0	4	2	1	1	2
#8	0	3	3	2	1	1

Preventive administration of ISE significantly improved the neurological scores of tMCAO mice compared to the vehicle-treated group (Kruskal–Wallis: *H* = 34.19, *p* < 0.0001, followed by Dunn’s multiple comparisons test. Post hoc comparisons: 50 mg/kg, *p* < 0.05 vs. the vehicle-treated tMCAO group; 100 mg/kg, *p* < 0.05 vs. the vehicle-treated tMCAO group; Clopidogrel, *p* > 0.05 vs. vehicle controls. *n* = 8 mice per group).

**Table 2:** Therapeutic effects of ISE on neurological deficit scores in tMCAO mice.

Group	Sham	Vehicle	ISE (50 mg/kg)
#1	0	3	1
#2	0	3	1
#3	0	3	1
#4	0	3	2
#5	0	2	2
#6	0	4	1
#7	0	3	1
#8	0	3	1

Therapeutic treatment with ISE (50 mg/kg) significantly ameliorated neurological deficits in tMCAO mice compared with the vehicle-treated group (Kruskal–Wallis: *H* = 21.65, *p* < 0.0001, followed by Dunn’s multiple comparisons test. Post hoc comparisons: 50 mg/kg, *p* < 0.05 vs. the vehicle-treated tMCAO group. *n* = 8 mice per group).

**Table 3:** Primer sequences used for qRT-PCR.

Gene	Forward Primer (5′–3′)	Reverse Primer (5′–3′)
iNOS	TCCAGGATGAGGACATGAGCAC	GAACGTCACACACCAGCAGGTTA
IL-1β	GCAACTGTTCCTGAACTCAACT	ATCTTTTGGGGTCCGTCAACT
TNF-α	CAGGCGGTGCCTATGTCTC	CGATCACCCCGAAGTTCAGTAG
IL-10	GCTCTTACTGACTGGCATGAG	CGCAGCTCTAGGAGCATGTG
YM1	CAGGGTAATGAGTGGGTTGG	CACGGCACCTCCTAAATTGT
Arg1	GCAGAGGTCCAGAAGAATGG	AGCATCCACCCAAATGACAC
GAPDH	AGGTCGGTGTGAACGGATTTG	TGTAGACCATGTAGTTGAGGTCA

## Data Availability

All data included in this study are available from the corresponding author upon reasonable request.

## Supplementary Materials

The following supporting information can be downloaded at: https://www.mdpi.com/article/10.3390/md24090328/s1, Figure S1: ISE had no significant effect on primary microglia viability after 24 h of incubation. Figure S2: Validation of LRP1 siRNA knockdown efficiency in primary microglia.
