# Supplementary material for: Isaridin E Attenuates Ischemic Brain Injury in Mice by Concurrently Reducing Platelet Hyperactivity and Microglial Neuroinflammation Associated with LRP1
Source: Mar Drugs. 2026 Sep 20;24(9):328. doi: 10.3390/md24090328 (PMC13608163; doi:10.3390/md24090328)
Supplement: Supplementary file 1 [file marinedrugs-24-00328-s001.zip › marinedrugs-4517508-supplementary.pdf]

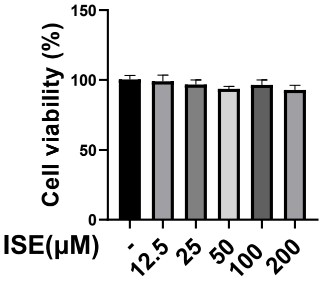

Figure S1. ISE had no significant effect on primary microglia viability after 24 h of incubation.

Cell viability of primary microglia was assessed by the CCK-8 assay. Primary microglia were treated with ISE (12.5, 25, 50, 100 or 200 μM) for 24 h. All data are shown as mean ± SEM. (n = 5). $p > 0.05$.

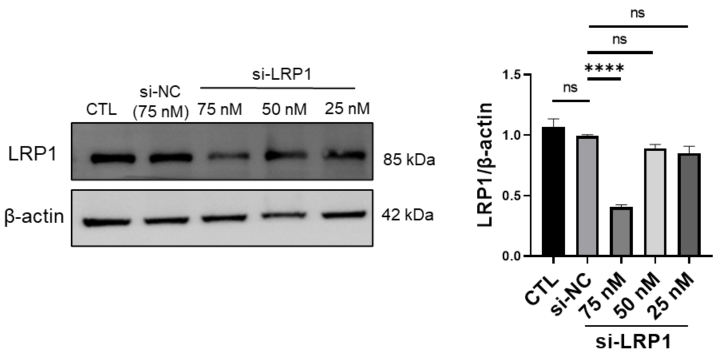

Figure S2. Validation of LRP1 siRNA knockdown efficiency in primary microglia.

After transfection with LRP1 siRNA (si-LRP1) / NC siRNA (si-NC) for 48 h, LRP1 protein levels in primary microglia were assessed by Western blot and quantified (n = 3). All data are shown as mean ± SEM. *ns*: $p > 0.05$, **** $p < 0.0001$.
